# Analysis of the application of functional near-infrared spectroscopy in acupuncture research: a review

**DOI:** 10.3389/fneur.2025.1644010

**Published:** 2025-09-19

**Authors:** Jili Xu, Xing Tang, Xiaotong Liu, Yi Wang, Jie Wu

**Affiliations:** ^1^College of Acupuncture and Tuina, Chengdu University of Traditional Chinese Medicine, Chengdu, China; ^2^Preventive Treatment Department, Chengdu Affiliated Hospital of Traditional Chinese Medicine, Chengdu, China

**Keywords:** acupuncture, fNIRS, brain function, hyperscanning, neuroimaging

## Abstract

Acupuncture, as an effective external treatment method, has been widely used in various clinical diseases, but the research on its mechanism still needs to be further deepened. Functional near-infrared spectroscopy (fNIRS) technology, as an emerging non-invasive neuroimaging technique, has the advantages of high timeliness, wide application scenarios, good portability and low cost. Currently, it is widely used in the mechanism research of acupuncture. This paper reviewed all the studies on the application of fNIRS in acupuncture, analyzed and summarized the current application status of fNIRS technology in acupuncture research. Firstly, we elaborated on the working principle of fNIRS and highlighted its advantages in acupuncture research and application. Secondly, we detailed the specific applications of fNIRS in acupuncture research. Finally, we discussed the existing problems of fNIRS in acupuncture research at present and the improvement directions for future research.

## Graphical Abstract

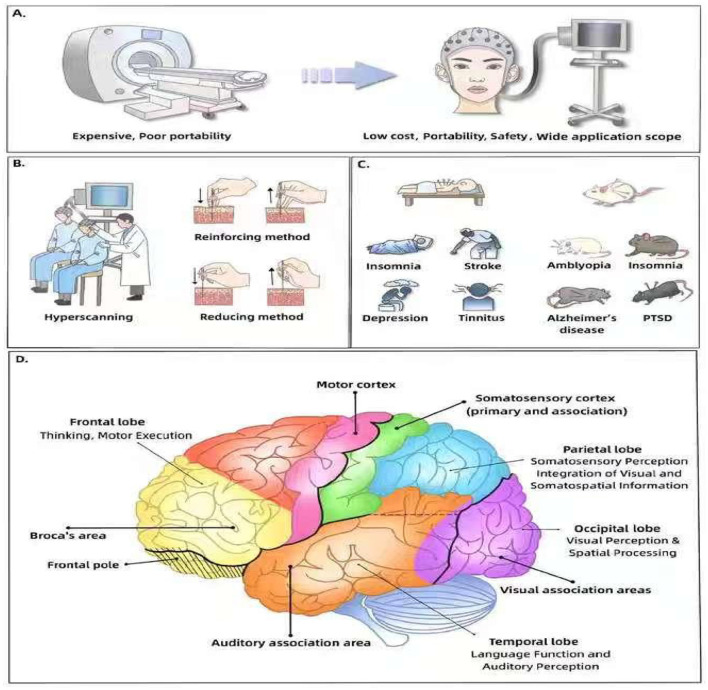

## 1 Introduction

Acupuncture is a non-pharmaceutical therapy based on the theory of Zang-Fu organs and meridians in traditional Chinese medicine. It has the functions of unblocking meridians, regulating qi and blood, and supporting the body's resistance and eliminating pathogenic factors. It can regulate human functions in multiple systems and at multiple levels, improve brain function, relieve pain (1), alleviate aging (2), and promote human recovery (3). Acupuncture, as an effective external treatment method, has the characteristics of a wide disease spectrum, simple application, economy and safety. However, the full understanding of its mechanism remains elusive. However, the mechanism of acupuncture treatment remains unclear, which limits its wide application in clinical practice. Research on the mechanism of acupuncture can help us gain a deeper understanding of its essence of action and the key factors for improving its efficacy.

As the advanced life center, the brain requires all kinds of life activities to be coordinated and exert their effects. Acupuncture stimulates the meridians and acupoints, eliciting sensations like pain and numbness, which are perceived by the brain, triggering corresponding changes (4). The regulatory effect of acupuncture on the human body needs to be integrated and processed by the brain to take effect. By making use of neuroimaging tools and methods, we can better understand the structure and function of the brain, and thereby study the working mechanism of the brain under physiological and pathological conditions. The development of neuroimaging has opened up new avenues for acupuncture research. At present, the main neuroimaging tools include fMRI, EEG, PET, fNIRS (5), etc. These several tools have been widely carried out in acupuncture research (6–9). Currently, fNIRS has become a hot spot in acupuncture research. Recent fNIRS studies (10) have shown that acupuncture exerted therapeutic effects by activating or inhibiting specific brain regions and regulating blood oxygen metabolism. In different diseases, the brain regions stimulated by acupuncture and the resulting effects vary; for the same disease, although the design schemes of different studies may differ, acupuncture can elicit similar cortical responses in the brain. So far, no one has discussed the acupuncture research using fNIRS. Therefore, we have reviewed all the fNIRS studies on acupuncture conducted and made a classification summary to better understand the brain neural mechanism by which acupuncture works.

## 2 The imaging principle of fNIRS and its advantages in acupuncture research

When the brain is functionally active, it is accompanied by the electrophysiological activities of neurons. Local neurons' electrical activities consume more glucose and oxygen (11), thereby enhancing local blood flow and oxygen levels, which subsequently boosts the concentration of oxygenated hemoglobin (Oxy-Hb) and reduces that of deoxygenated hemoglobin (Deoxy-Hb) (12). The changes in the concentrations of Oxy-Hb and Deoxy-Hb and their locations can indirectly reflect the intensity and location of functional activities in response brain tissue (13). In 1977, near-infrared spectroscopy (NIRS) research found that infrared light with wavelengths ranging from 700 to 900 nm has a high penetrating power for brain tissue (14). Meanwhile, blood hemoglobin has good absorption and scattering characteristics for near-infrared light with wavelengths ranging from 600 to 900 nm. Since then, NIRS technology has begun to be applied in the study of brain neural function. fNIRS is a technique that indirectly monitors neuronal activities based on the above neurovascular coupling principle. Its imaging consists of four parts: fNIRS signal acquisition, fNIRS signal preprocessing, feature extraction of blood oxygen concentration in brain tissue, and signal presentation (15).

In recent years, fNIRS has become a mature imaging tool in neuroscience research (16). The temporal resolution of functional magnetic resonance imaging (fMRI) and positron emission tomography (PET) is low (17, 18), making it impossible to conduct real-time and dynamic monitoring of changes in brain activity during acupuncture manipulation. At the same time, their experimental environment are extremely demanding. The fNIRS has a high temporal resolution, enabling it to reflect immediate changes in brain function. Its real-time monitoring capability is even more applicable (making it more suitable for conducting research on the immediate effects of acupuncture). Compared with electroencephalography (EEG), fNIRS has a higher spatial resolution (19) and can monitor the metabolic responses of the local cerebral cortex during acupuncture operations (20). The fNIRS equipment is lightweight and flexible, thus having high portability (more friendly for conducting tests on patients with motor dysfunction such as stroke and Parkinson's disease), with low requirements for the experimental environment, simple and quick operation, a large number of probes, capable of conducting research between two or even multiple individuals, and has a wider application scenario for acupuncture studies compared to fMRI and PET. The fNIRS equipment uses flexible optical fiber sensors that can be fixed on the head, allowing the subjects to perform a certain degree of voluntary movement. Compared with fMRI and PET, it has greater resistance to motion artifacts (21). At the same time, the cost of fNIRS is even lower. During acupuncture research, subjects may exhibit movement due to fear or pain, making it more suitable for conducting acupuncture studies. From the above, it can be seen that fNIRS has several unique advantages in acupuncture research, including high timeliness, low cost, wide application scope, good portability, and strong resistance to motion artifacts.

## 3 Review method

**Figure 1:**
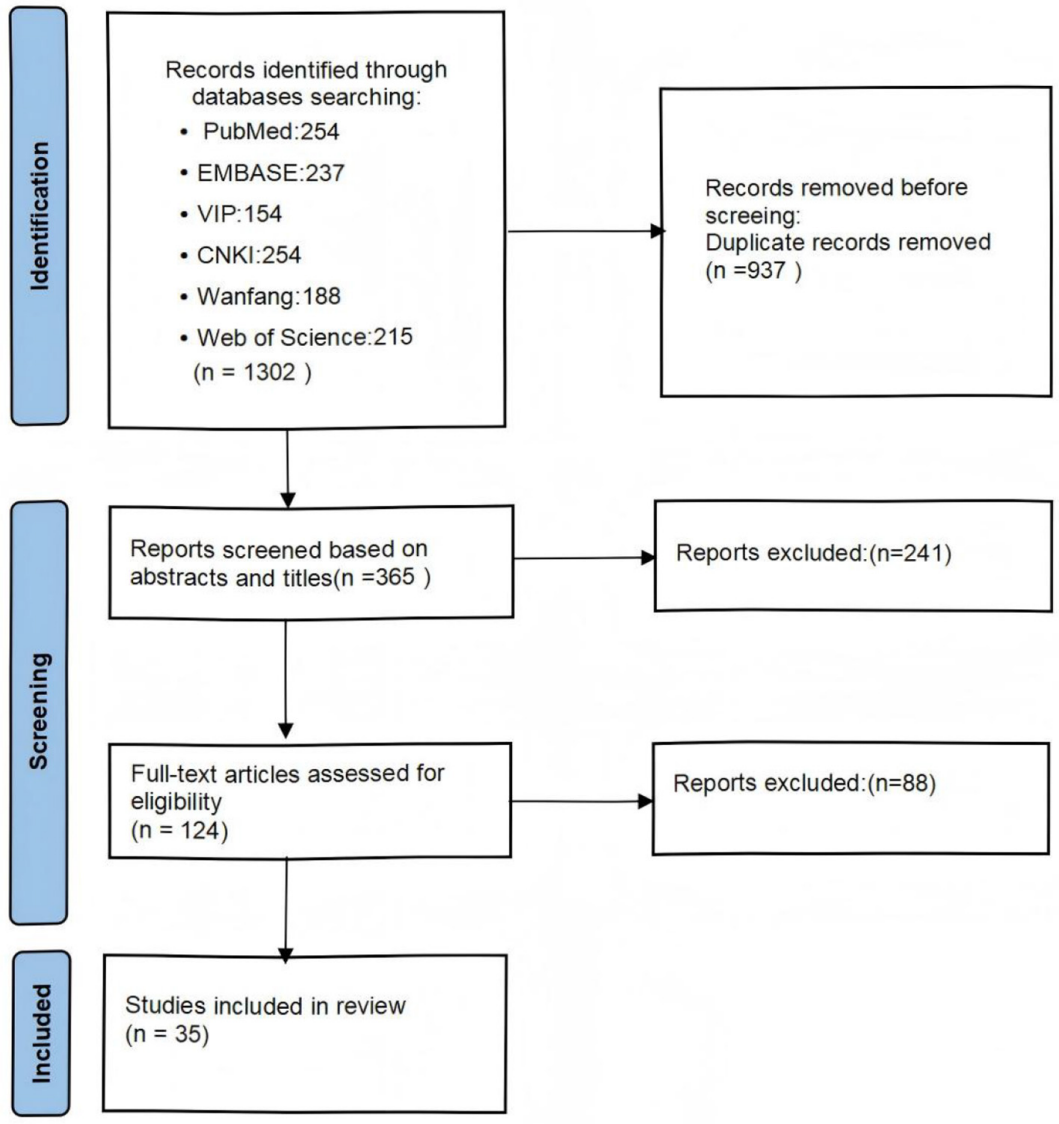
Literature screening flowchart.

We conducted a comprehensive search of five databases: PubMed, China National Knowledge Infrastructure (CNKI), Web of Science, Wanfang Database, and China Science and Technology Journal Database (VIP). The search period was set from January 2015 to April 2025. The search terms for both Chinese and English literature were “(acupuncture OR acupuncture treatment)” AND “(fNIRS OR functional near-infrared spectroscopy).” The studies must be articles published in English or Chinese. The intervention methods were limited to acupuncture or acupuncture combined with other treatments, and case reports, reviews, and trial protocols were excluded. Detailed search strategy, inclusion and exclusion criteria for the literature can be found in the Supplementary material. The flowchart of literature screening is given in Figure 1.

## 4 Results

We identified a total of 35 fNIRS studies involving acupuncture. Among them, 31 were completed in China, one was being carried out for Australia, one was conducted in Japan, and three were completed in South Korea. Among the retrieved literature, there were four animal experiments using rats as the subjects, and 31 clinical studies using humans as the subjects. In the clinical studies, six were conducted under physiological conditions of acupuncture, and 25 were conducted under pathological conditions of acupuncture. The main characteristics of studies were shown in Tables 1–3.

**Table 1:** Research on fNIRS acupuncture under normal physiological conditions.

**Study**	**Characteristics of participants**		**Intervention type**		**fNIRS parameter**	**fNIRS paradigm**	**Study brain regions**	**Main findings**
	**Experimental group**	**Control group**	**Experimental group**	**Control group**				
**ARRM**								
Qu et al. (24) (China)	Healthy participants *n* = 33 age: 20.70 ± 2.34 years	/	SSH and TTL on *Quchi* (LI 11)	/	3.91 HZ; 36-channels	Resting-state 300 s Acupuncture 300 s	SMC and PFC	The results found that SSH and TTL could elicit significant cerebral responses, respectively, but there was no difference between them
Cao et al. (25) (China)	Healthy participants *n* = 31 age: 20.70 ± 2.34 years	/	Acupuncture *Quchi* (LI 11) using three different ARRMs	/	3.09 HZ; 36-channels	Resting-state 300 s acupuncture *Quchi* acupoint (LI11) 2 min 10 s	PFC, bilateral somatosensory cortex	The reinforcing manipulation activated the bilateral DLPFC, the left S1, and the right S2; the reducing manipulation deactivated the bilateral DLPFC; the even reinforcing-reducing manipulation deactivated the bilateral DLPFC, FP, the right M1, the bilateral S1, and the bilateral S2
***Deqi*** **and manipulations of acupuncture**								
Song et al. (27) (China)	Healthy participants *n* = 29 age: 25.03 ± 0.66	Healthy participants *n* = 29 age: 25.03 ± 0.66	Acupuncture *Shousanli* (LI 10) acupoint *deqi* The twisting Angle was 180 to 360 °, and the frequency was 120 times per minute	Acupuncture *Shousanli* (LI 10) acupoint no *deqi* The twisting Angle was 60–90 ° and the frequency was 60 times per minute	11 HZ; 46-channels	Block design Resting-state 60 s acupuncture 300 s	Bilateral frontal lobes, parietal lobes and temporal lobes	*Deqi* could activate the bilateral frontal lobes of the subjects, enhanced the FC between different brain regions, and the brain functional responses of the subjects might help objectively characterize the effect of *deqi*
Si et al. (28) (China)	Healthy participants *n* = 20 (12 M, 8 F), age: 23.9 ± 1.5 years	/	Acupuncture *Hegu* (LI4) acupoint	/	9 HZ; 48-channels	Quasi-experimental design	PFC, MC, BA8	During acupuncture manipulation, the concentrations of Oxy-Hb in the PFC and motor cortex decreased, and their functions were significantly inhibited. The network's efficiency improved by acupuncture manipulation
Fernandez Rojas et al. (30) (Australia)	Healthy participants *n* = 11 (12 M, 8 F) Age: 23.9 ± 1.5 years	/	Acupuncture *Hegu* (LI4) acupoint	/	10 Hz; 24-channels	Block design; acupuncture 40 s	SI	During the needle insertion and needle removal, the hemodynamic response showed strong activation, and rotation period the hemodynamic response decreased.
**Hyperscanning**								
Li et al. (34) (China)	Healthy participants *n*=31; acupuncturist *n* = 1 age: 20.710 ± 2.341 years	Healthy participants *n* = 33; acupuncturist *n* = 1 age: 19.971 ± 1.360 years	VA on *Quchi* (LI 11) acupoint	Sham acupunctureon *Quchi* (LI 11) acupoint	3.91 HZ; 36-channels	Baseline (5 min) and acupuncture manipulation (2 min)	SMC and PFC	INS in the PFC of “patient”– acupuncturist dyad was significantly increased during verum but not sham acupuncture stimuli, and positively correlated with the needling sensations of “patients.”

PFC, prefrontal cortex; SI, primary somatosensory cortex; S2, secondary somatosensory cortex; MC, motor cortex; INS, interpersonal neural synchronization; Oxy-Hb, oxygenated hemoglobin; Deoxy-Hb, deoxygenated hemoglobin; Total-Hb, total hemoglobin; DLPFC, dorsolateral prefrontal cortex; MI, primary motor cortex; TTL, “Toutianliang” reducing method; SSH, “Shaoshanhuo” reinforcing method; SMC, sensorimotor cortex; BA 8, Brodmann area 8; FC, functional connection.

**Table 2:** Research on fNIRS acupuncture under pathological conditions.

**Study**	**Characteristics of participants**		**Intervention type**		**fNIRS parameter**	**fNIRS paradigm**	**Study brain regions**	**Main findings**
	**Experimental group**	**Control group**	**Experimental group**	**Control group**				
**Insomnia**								
Xu (36) (China)	Patients with insomnia *n* = 29 (10 M, 19 F) Age: 41.34 ± 12.28 years	Healthy participants *n* = 30 (12 M, 18 F) Age: 41.34 ± 12.28 years	Acupuncture at Baihui (GV20), Neiguan (PC6), Shenmen (HT7), Sanyinjiao (SP6), and Zhaohai (KI6)	Acupuncture at Baihu (GV20) i, Neiguan (PC6), Shenmen (HT7),Sanyinjiao (SP6) and Zhaohai (KI6)	6.25 Hz; 46-channels	Facial expression recognition task	Bilateral frontal and parietal	Acupuncture improved the function of the related brain regions by regulating the blood oxygen concentration in the brain lobes, orbitofrontal cortex and parietal lobe
Wang et al. (37) (China)	Young insomnia patients *n* = 10 (3 M, 7 F) Age: 18–35 years Middle aged insomnia patients *n* = 12 (2 M, 10 F) Age: 35–50 years Elderly insomnia patients *n* = 8 (3 M, 5 F) Age: 50–65 years	/	Acupuncture therapy three times/week, 2 weeks	/	6.25 Hz; 46-channels	Event Experimental Design: positive, neutral, negative Sexual emotion face test	Frontal lobe	The young group showed a decrease in the Oxy-Hb concentration in the frontal under the condition of “Yang,” which includes both positive and negative emotional faces. There was no significant difference in Oxy-Hb concentration in young group under the condition of “Yin,” which are neutral faces. The Oxy-Hb concentration of the middle-aged group decreased under the “Yang” condition of the negative emotional faces, while increased under the “Yin” condition of the neutral faces. The concentration of Oxy-Hb of the elderly group increased under the “Yin” condition of neutral face
**Mental disorders**								
Chen (41) (China)	Depressed patients *n* = 8 (3 M, 5 F) Age: 48.13 ± 9.75 years	Depressed patients *n* = 8 (2 M, 6 F) Age: 47.9 ± 9.9 years	Acupuncture treatment was administered three times a week for a total of 24 times over 8 weeks	Escitalopram oxalate tablets were orally administered for 8 weeks	/	Emotional image processing task	DLPFC	Both acupuncture and Western medicine increased the percentage of Oxy-Hb in the DLPFC of patients with depression, but the change of acupuncture was more significant at the end of the first week
Miao (42) (China)	Depressed patients *n* = 8 (3 M, 5 F); Age: 47.62 ± 6.72 years	Depressed patients *n* = 8 (2 M, 6 F) Age: 48.12 ± 7.74 years	Acupuncture treatment was administered three times a week for a total of 24 times over 8 weeks	Escitalopram oxalate tablets were orally administered for 8 weeks	250 Hz; 4-channels	Emotional image processing tasks, working memory tasks, and brain working ability index tasks	Prefrontal lobe	The concentrations of Oxy-Hb in the prefrontal lobe corresponding to the three types of emotional images in both groups of patients increased after treatment. Among them, there were statistically significant differences in the prefrontal Oxy-Hb concentration corresponding to the sad pictures between the two groups at the 1st, 2nd, 4th, and 8th levels, while there were no statistically significant differences in the other two types of emotional pictures
Chen (43) (China)	Depressed patients *n* = 12 (1 M, 11 F); Age: 41.66 ± 17.43 years	Healthy people *n* = 12 (3 M, 9 F) Age: 40.25 ± 11.99 years	Acupuncture treatment was administered three times a week for a total of 12 times over 8 weeks	/	250 Hz; 4-channels	Detection of Oxy-Hb, deoxy-Hb, and total-Hb concentrations	Left dorsolateral prefrontal lobe	Acupuncture could increase the level of Oxy-Hb in the left dorsolateral prefrontal lobe of the brain in patients with depression and reduce the level of Deoxy-Hb, thereby improving the inhibitory state of the patient's brain. In healthy individuals, the left dorsolateral prefrontal lobe of the brain showed no significant response to acupuncture, suggesting that whether the brain was injured or not was an important factor affecting whether there was a response to acupuncture
Wong et al. (44) (China)	Depressed patients *n* = 8; Age: 44.8 ± 10.3 years	Depressed patients *n* = 12 Age: 50.9 ± 11.1 years	Acupuncture combined antidepressants treatment for 3 weeks	Antidepressants monotherapy for 3 weeks	7.81 Hz; 20-channels	Resting state	DLPFC	Acupuncture combined with antidepressants compared to the sole use of antidepressants had a more significant reduction in depressive symptoms and a significantly stronger rsFC in the DLPFC after 3 weeks of treatment
Xiang (45) (China)	Patients with generalized anxiety disorder *n* = 12 (2 M, 10 F) Age: 45.42 ± 13.41 years	Healthy participants *n* = 12 (3 M, 9 F) Age: 40.25 ± 11.99 years	Acupuncture at Sishencong (EX-HN1), Zhongwan (RN12), Qihai (RN6), Hegu (LI4),Taichong (LR3) Zusanli (ST36) Sanyinjiao (SP6) and Shenmen (HT7)	Acupuncture at Sishencong, Zhongwan, Qihai, Hegu, Taichong Zusanli Sanyinjiao and Shenmen	250 HZ	Block design; Acupuncture stimulation–acupuncture preparation–acupuncture stimulation	Left DlPFC	Acupuncture activated the left dorsolateral prefrontal lobe of patients with generalized anxiety disorder, presenting an immediate response characterized by elevated Oxy-Hb and decreased Deoxy-Hb. The improvement of generalized anxiety disorder by acupuncture may be related to the activation of the left DPFC
Tamai et al. (46) (Japan)	Patients with acute stress *n* = 13	Patients with acute stress *n* = 13	GV20 verum acupuncture	GV20 sham acupuncture	0.76 Hz; 16-channels	UKT task	PFC	VA had a faster inhibitory effect on subjective stress levels and promotes faster recovery than SA improved the stress caused by acute stress by maintaining the CBF of the left lateral ventral PFC
**Sequelae of stroke**								
Zhang et al. (56) (China)	Patients with hemiplegia after stroke *n*=19 (4 M, 19 F) Age: 60.9 ± 13.7 years	/	Acupuncture	/	10 Hz; 32-channels	Resting state (10 min) and during acupuncture treatment (10 min)	PFC and MC	Acupuncture could induce bilateral cerebral activation responses in hemiplegic stroke patients. Moreover, the cerebral cortex activation responses of patients with severe motor function impairment are more significant than those of patients with mild impairment under acupuncture, especially in the affected hemisphere
Ma (50) (China)	Patients with dysphagia after stroke *n* = 28; Age: 68.00 (59.0, 70.0) years	Patients with dysphagia after stroke Acupuncture group *n* = 29 Age: 66.00 (62.5, 75.0) years tDCS Group: *n* = 29 Age: 67.00 (56.0, 75.5) years	Conventional swallowing rehabilitation treatment was administered once a day for a total of 14 days	Acupuncture group: Acupuncture combined with swallowing rehabilitation training for 14 days, with acupuncture once a day tDCS group: tDCS stimulation combined with swallowing rehabilitation training for 14 days, with acupuncture once a day	11 Hz; 47-channels	Swallowing task	The precentral and posterior central gyrus of the primary sensorimotor cortex, PMC, BA6	The activation intensity and FC of the brain regions of interest in the acupuncture group and the group were significantly enhanced compared with the control group
Jiang (51) (China)	Patients with dysphagia after stroke *n* = 24 (15 M, 9 F); Age: 64.08 ± 5.57 years	Patients with dysphagia after stroke *n* = 24 (16 M, 8 F) Age: 63.25 ± 8.17 years	The treatment of penetrating swallowing acupuncture combined with swallowing rehabilitation training lasted for 14 days	Conventional swallowing rehabilitation treatment for a total of 14 days	10 Hz; 16-channels	Resting state (90 s) and swallowing task state (90 s)	The frontal and parietal cortex	The “penetrating swallowing needle technique” increased the FC in the brain regions, especially the S1 and the M1 as well as the PMC and SMC
Zhang et al. (53) (China)	Patients with upper limb movement disorders after stroke *n* = 20 (15 M, 5 F); Age: 55.95 ± 16.98 years	Patients with upper limb movement disorders after stroke *n* = 20 (10 M, 10 F) Age: 59.8 ± 11.63 years	Auricular acupuncture combined with conventional rehabilitation training treatment, among which auricular acupuncture treatment was conducted once a day for a total of 10 sessions	False auricular acupuncture combined with conventional rehabilitation training treatment, among which auricular acupuncture treatment was conducted once a day for a total of 10 treatments	10 Hz; 38-channels	Resting-state design (600 s)	M1	After auricular acupuncture treatment, compared with the control group, the peak MEP in the M1 area of the brain in the auricular acupuncture group was significantly increased, the content of Oxy-Hb was significantly increased, and the activation effect was significant
Tang et al. (52) (China)	Patients with lower extremity motor dysfunction after stroke *n* = 24, randomly divided into rehabilitation group *n* = 12 (11 M, 1 F) (Age: 51.91 ± 4.63 years) and acupuncture combined with rehabilitation group *n* = 12 (11 M, 1 F; age: 53.50 ± 6.00 years)	Healthy people *n* = 10 (9 M, 1 F) Age: 46.10 ± 11.00 years	Rehabilitation group: rehabilitation training Acupuncture combined with rehabilitation group: scalp acupuncture combined with rehabilitation training; five times/week, 4 weeks	/	10 Hz; 24-channels	Walking task	SMC, PMC and sensory motor cortex	Acupuncture with rehabilitation therapy could significantly improve the lower limb motor function and asymmetrical activation of sensory motor cortex in stroke patients. The recovery of lower limb motor function might be related to the enhanced activation of affected PMC
Zheng et al. (55) (China)	Hemiplegic patients with ischemic stroke *n* = 29 (14 M, 15 F); Age: 42.17 ±5.93 years	Hemiplegic patients with ischemic stroke *n* = 29 (15 M, 14 F) Age: 41.89 ±5.91 years	Staged acupuncture treatment was administered once a day for a total of 35 times	Conventional acupuncture treatment was administered once a day for a total of 35 times	20 Hz; 41-channels	Oxy-Hb	PMC, premotor cortex, auxiliary motor area, FP, DLPFC and BA	Staging acupuncture could improve motor function, improve ability of daily living, reduce nerve function defect and activate brain nerve function in patients with ischemic stroke
Zheng et al. (54) (China)	Patients with cerebral infarction *n* = 29 (14 M, 15 F) Age: 40.9 ± 8.1 years *n* = 29 (14 M, 15 F) Age: 40.9 ± 8.1 years	Patients with cerebral infarction *n* = 29 (15 M, 14 F) Age: 40.3 ± 8.9 years	Acupuncture combined with hydrotherapy, once a day	Acupuncture treatment, once a day	20 Hz; 41-channels	Oxy-Hb	PMC or cortex, auxiliary motor area, FP, DLPFC, BA	Acupuncture combined with hydrotherapy rehabilitation technology can improve the daily living ability and balance function of patients with cerebral infarction, enhance motor function, reduce GFAP and sdLDL levels, activate brain nerve function, and improve treatment effectiveness
Lin et al. (57) (China)	Patients with aphasia after stroke *n* = 32 (27 M, 5 F)	Patients with aphasia after stroke *n* = 32 (23 M, 9 F)	Scalp acupuncture combined with speech and language training	Speech and language training (SLT)	10.101 Hz; 110-channels	Resting-state Oxy-Hb		Oxy-Hb D-values were significantly greater in the left FP area and right superior temporal gyrus compared to the control group. Among non-global aphasia patients, acupuncture improved comprehension and naming tasks, with lower Oxy-Hb in the right visual association cortex and angular gyrus. In global aphasia patients, improvements were seen in the Repetition scores, with higher OxyHb in the right inferior prefrontal gyrus
**Disorders of consciousness**								
Liu et al. (58) (China)	Patients with prolonged disorder of consciousness *n* = 14 (10 M, 4 F) Age: 62.0 ± 15.6 years	Patients with prolonged disorder of consciousness *n*=14 (8F6F) Age: 48.9 ± 20.2 years	Acupuncture at the *Neiguan* (PC6) acupoint	SA at the sham point	13.33 Hz; 20-channels	Resting state; experimental paradigm including needle insertion, needle insertion and needle removal	DLPFC, M1 and S1	Compared to the control group, a single session of acupuncture significantly tended to enhance rsFC in DLPFC-M1, DLPFC-M1, and S1-S1. And the activation level of the DLPFC (both sides) in the acupuncture group is significantly higher than those in SA group
Xin et al. (59) (China)	Patients with disorder of consciousness *n* = 16 (7 M, 9 F) 60.75 ± 19.74 years	/	Acupuncture at the Renzhong point	/	13.33 Hz; 48-channels	Block sequence of three tasks, including needle insertion, needle insertion and needle remova	PFC	Oxy-Hb in the PFC was increased during the acupuncture manipulation and declined during the resting state. Then, the connection strength of the left cerebral cortex was generally higher than that of the right
**MCI**								
hafoor et al. (60) (Korea)	MCI patients *n* = 12 (0 M, 12 F) Age: 61.58 ± 6.55 years	Healthy people *n* = 12 (0 M, 12 F) Age: 55.92 ± 7.65 years	Acupuncture therapy Twice a week, for a total of 24 times	/	7.81 Hz; 20-channels	Block design; resting state and working memory task state	PFC	In both the resting state and the task state, Oxy-Hb increased and FC was also enhanced
Khan et al. (61) (Korea)	MCI patients *n* = 11 (0 M, 11 F) Age: 61.58 ± 6.55 years	Healthy people *n* = 11 (0 M, 11 F) Age: 55.92 ± 7.65 years	Acupuncture therapy	/	7.81 Hz; 20-channels	Working memory task	PFC	Acupuncture therapy improved the hemodynamic response of patients. This statement is consistent with the cognitive performance of the participants monitored through the MoCA test scores. Furthermore, the results indicated that acupuncture increased the activation area and connectivity of patients with MCI
**Tinnitus**								
Yu et al. (62) (China)	Patients with bilateral tinnitus *n* = 18 (10 M, 8 F) age: 27–67 years	/	Acupuncture was performed every other day for a total of 10 times	/	10 Hz; 20-channels	Schematic diagram of the auditory stimulus paradigm	Temporal lobe	Acupuncture increased the concentration of oxygenated hemoglobin in the temporal lobe of tinnitus patients, and affected the activation of the auditory cortex.
Fan et al. (63) (China)	Patients with sensorineural tinnitus *n* = 14 (4 M, 10 F) Age: 48 ± 11.68 years	Healthy people *n*=14 (1 M, 13 F) Age: 26.86 ± 3.60 years	Acupuncture treatment for 1 month	/	20 Hz; 20-channels	Sound stimulation task	Temporal lobe	Acupuncture treatment for 1 month significantly increased the blood oxygen concentration in the temporal lobe
**Amblyopia**								
Wu (67) (China)	Children with anisometropic amblyopian *n* = 24 (13 M, 11 F) Age: 6.63 ± 0.13 years	Children with anisometropic amblyopian *n* = 23 (13 M, 8 F); Age: 6.52 ± 0.13 years	Acupuncture therapy	Transcutaneous electrical stimulation of acupoints	22-channels	Monocular visual stimulation task	V1; V2	When visual stimulation was received in one eye, children with anisometropic amblyopia had blood oxygen dysfunction in both the V1 and V2, and there was asymmetric involvement between the bilateral visual cortices. Both traditional acupuncture and percutaneous electrical acupoint stimulation intervention increased the Oxy-Hb content in the visual cortex of children with anisometropic amblyopia when they received visual stimulation in one eye, and at the same time improved the asymmetric involvement between the ipsilateral and contralateral visual cortices
Chai et al. (66) (China)	Patients with monocular anisometropic amblyopia *n* = 15 (7 M, 8 F) Age: 7–12 years	Healthy children *n* = 38 (18 M, 20 F) Age: 7–12 years	Acupuncture treatment combined with conventional treatment	/	22-channels	Visual stimulation task design	Prefrontal lobe, occipital lobe	The activation range of the visual cortex in patients with amblyopia was smaller than that in healthy subjects. During the baseline period and the high-frequency stimulation period, the visual cortex of the amblyopic eye was affected. The stratified blood oxygen response was weaker than that of healthy eyes and normal people. Acupuncture improved the hemodynamic response of the visual cortex
**PD**								
Jang et al. (70) (Korea)	Parkinson's patients *n* = 13 (10 M, 3 F) Age: 65.38 ± 7.81 years	Parkinson's patients *n* = 13 (7 M, 6 F) Age: 61.46 ± 8.33 years	Acupuncture combined with conventional treatment for 4 weeks, with acupuncture twice a week	Conventional treatment lasts for 4 weeks	4.17 Hz	Walking task	Movement and the prefrontal cortex	In the acupuncture combined with conventional treatment group, there was a significant increase in hemoglobin levels in the prefrontal lobe and auxiliary motor areas. The level of hemoglobin oxygenation showed a significant positive correlation between the prefrontal cortex and swing time

FC, functional connectivity; FP, frontal polar; PFC, prefrontal cortex; MI, primary motor cortex; MC, motor cortex; SI, primary somatosensory cortex; S2, secondary somatosensory cortex; PMC, premotor cortex; SMC, supplementary motor cortex; SMA, supplementary motor area; DLPFC, dorsolateral prefrontal cortex; CBF, cerebral blood flow; PD, Parkinson's disease; Oxy-Hb, oxygenated hemoglobin; Deoxy-Hb, deoxygenated hemoglobin; Total-Hb, total hemoglobin; V1, primary visual cortex; V2, secondary visual cortex; rsFC, resting-state functional connectivity; GFAP, glial fibrillary acidic protein; sdLDL, small dense low-density lipoprotein; UKT, the Uchida–Kraepelin test; BA, Brodmann area; MEP, motor evoked potential; MCI, mild cognitive impairment; AD, Alzheimer's disease; MoCA, montreal cognitive assessment.

**Table 3:** Animal acupuncture research.

**Study**	**Characteristics of rats**		**Intervention type**		**fNIRS parameter**	**fNIRS paradigm**	**Study brain regions**	**Main findings**
	**Modeling group**	**Blank group**	**Modeling group**	**Blank group**				
Zhang (71) (China)	PTSD rats, Model group (*n* = 12) Grasping group (*n* = 12) Paroxetine group (*n* = 12) Acupuncture group (*n* = 12)	Healthy rats (*n* = 12)	Model group: replication model Grasping group: replicate the model and fix it using the same grasping method as each treatment group Paroxetine group: replicate the model and receive treatment by oral administration of paroxetine hydrochloride solution. Acupuncture group: replicate the model and acupuncture the four acupoints “Baihui” (GV20), “Neiguan” (PC6), “Shenmen” (HT7) and “Taichong” (LR3)	Blank group: no intervention	Resting state (180 s)	Detection of Oxy-Hb, deoxy-Hb and Total-Hb concentrations in the cerebral cortex of rats in each group	Prefrontal lobe, occipital lobe, hippocampus, amygdala	“Dispering liver and regulating spiit” god stitch can obviously improve the abnormal cortex ofrats oxygenated hemoglobin, showed that acupuncture treatment effectively; “Dispering liver and regulating spiit” god stitch intervention PTSD main central mechanism and prefrontal cortex, hippocampus, amygdala brain regions associated plasticity
Ma et al. (72) (China)	Right eye deprivation rats Model group (*n* = 12) LDME group (*n* = 12) Early acupuncture group (*n* = 12) Mid-term acupuncture group (*n* = 12) Late acupuncture group (*n* = 12)	Healthy rats (*n* = 10)	Model group: no processing was carried out LDME group: administer LDME at a dose of 40 mg/kg was administered by gavage starting from the third day Early acupuncture group: acupuncture began on the third day Mid-term acupuncture group: acupuncture began on the 12th day Late acupuncture group: acupuncture began on the 21st day, each of the three acupuncture groups received acupuncture for 9 days, once a day	/	Resting state	The concentrations of Oxy-Hb, Deoxy-Hb, and Total-Hb in the dominant columns of the visual cortex	Area 17 of the visual cortex	The functions of the ocular dominance columns in rats' contralateral (left) visual cortex are significantly inhibited after unilateral (right eye) deprivation, and the functions of the ocular dominance columns in rats' ipsilateral (right) visual cortex are reorganized abnormally. Early acupuncture can effectively regulate the “shift” and reorganization of visual column after visual deprivation
Ye et al. (73) (China)	Sleep deprivation model rats, Model group (*n* = 10) grabbing group (*n* = 10) Western medicine group (*n* = 10) Acupuncture group (*n* = 10) Sham acupuncture group (*n* = 10)	Healthy rats (*n* = 10)	Model group: no intervention Grasping group: the grasping treatment was started on the second day and the grasping method, strength and duration were the same as the Western medicine group and acupuncture group. Western medicine group: gavage with eszopiclone (0.2 mg/kg) starting on day 2 after model replication, 1 time/day for 7 days. Acupuncture group: acupuncture was performed on Baihui (GV20), Neiguan (PC6), Shenmen (HT7) and Taichong (LR3), one time/day for 7 days. Sham acupuncture group: rats were selected from the tail root and the upper, middle and lower 1/3 of the tail, 16 each point was operated for one time/day, and treated for 7 days.	Blank group: no intervention	Resting state (180 s)	The concentrations of Oxy-Hb, Deoxy-Hb and Total-Hb	Prefrontal lobe and occipital lobe	The needling method for soothing the liver *Qi* and regulating mind could improve the abnormal behavior of insomnia rats with liver *Qi* stagnation, and its effect on the improvement of abnormal mood caused by insomnia with liver *Qi* stagnation is better than that of Western Medicine, and the mechanism of action may be related to the regulation of blood oxygen metabolism in the prefrontal and occipital lobes of the cerebral cortex by acupuncture
Zhang et al. (74) (China)	AD model rats Model group (*n* = 10) model group, Donepezil hydrochloride group (*n* = 10), acupuncture group (*n* = 10)	Healthy rats (*n* = 10) Sham operation group (*n* = 10)	Model group: no intervention Donepezil hydrochloride group: donepezil hydrochloride solution at a dose of 0.125 mg/ml (0.45 mg/kg) was administered by tube feeding once a day for 7 consecutive days as one course of treatment, with a total of four courses Acupuncture group: acupuncture at “Baihui” (GV20), “Sishencong” (EX-HN1), “Neiguan” (PC6), “Shenmen” (HT7) and “Sanyinjiao” (SP6),the treatment lasts for 6 days, followed by 1 day of rest. 7 days constitute one course of treatment, totaling four courses.	Blank group: no modeling and any intervention were performed Sham operation group: sham operation group: only modeling surgery was performed and no modeling drugs were injected	Resting state (180 s)	Detection of Oxy-Hb, Deoxy-Hb, and Total-Hb concentrations in the cerebral cortex of rats	/	Acupuncture with the “Yizhi Tiaoshen” acupoint formula and donepezil hydrochloride can improve the learning and memory function of AD rats, and its mechanism may be related to improving blood oxygen metabolism in the prefrontal and parietal regions and protecting neuronal function

FC, functional connectivity; FP, frontal polar; PFC, prefrontal cortex; MI, primary motor cortex; MC, motor cortex; SI, primary somatosensory cortex; S2, secondary somatosensory cortex; PMC, premotor cortex; SMC, supplementary motor cortex; SMA, supplementary motor area; DLPFC, dorsolateral prefrontal cortex; CBF, cerebral blood flow; Oxy-Hb, oxygenated hemoglobin; Deoxy-Hb, deoxygenated hemoglobin; Total-Hb, total hemoglobin; AD, Alzheimer's disease; PTSD, post-traumatic stress disorder; LDME, Aladdin industrial corporation.

## 5 Acupuncture studies of fNIRS in physiological state

Studies on acupuncture under physiological conditions mainly explored the effects of acupuncture on the brain functions of healthy subjects through fNIRS. Summarizing the content, it mainly covered three aspects: acupuncture reinforcing-reducing manipulation (ARRM), *deqi* and manipulations of acupuncture, and hyperscanning between the doctor and the patients.

### 5.1 ARRM

ARRM is a key factor influencing the clinical efficacy of acupuncture. As one of the necessary procedures in traditional acupuncture techniques, it plays a crucial role. In clinical practice of acupuncture, the reinforcing manipulation is particularly suitable for patients presenting with deficiency symptoms, as it aims to replenish the body's vital energy and improve circulation (22). Conversely, the reducing manipulation is more appropriate for those with excess symptoms, as it seeks to eliminate excess energy and alleviate blockages (23). The application of neuroimage techniques to clarify the mechanism of acupuncture has become a hot topic in acupuncture research. fNIRS, as a gradually mature imaging tool, has been applied in the study of the mechanism of ARRM. At present, there are a total of two fNIRS studies related to ARRM.

Qu et al. (24) conducted the first study on the mechanism of different acupuncture reinforcing and reducing manipulations (ARRMs). The research focused on two classic compound ARRMs: “Shaoshanhuo” reinforcing method (SSH) and the “Toutianliang” reducing method (TTL). The results revealed that during SSH, the bilateral primary motor cortex (MI), left primary somatosensory cortex (SI), and right somatosensory association cortex were activated in the subjects. In TTL, the bilateral prefrontal cortex (PFC) and the bilateral MI were activated, the functional connection (FC) between the PFC and SI, the left PFC and left MI were increased. However, the differences between the two were not significant in terms of cortical activation and brain FC.

Another study examined the responses of the cerebral cortex to three different types of simple ARRMs: lifting-thrusting reinforcing manipulation, lifting-thrusting reducing manipulation and reinforcing-reducing manipulation with lifting-thrusting (25). The results revealed that three types of ARRMs similarly induced the hemodynamic responses in the bilateral dorsolateral prefrontal cortex (DLPFC) and increased FC between the DLPFC and S1. Among them, the even reinforcing-reducing manipulation deactivated the bilateral DLPFC, the frontal polar (FP) area, the right M1, the bilateral S1, and the bilateral secondary somatosensory cortex (S2). The reinforcing manipulation activated the bilateral DLPFC, the left S1, and the right S2; the reducing manipulation deactivated the bilateral DLPFC. It indicated that the reducing manipulation and the reducing manipulation induced opposite hemodynamic responses in bilateral DLPFC and left S1. Bilateral DLPFC and left S1 were potential target brain regions for the differences in the effects of ARRMs. In conclusion, both studies have shown that different ARRMs had an impact on the activation of the cerebral cortex and its FC. As can be seen from Table 1, the two research trials have consistency in aspects such as subject selection, acupuncture point selection, and fNIRS task paradigm. However, the trial results are not completely consistent. Given the limited number of studies on ARRM at present, it is impossible to draw a definite conclusion. In the future, more rigorous studies on ARRM need to be designed and carried out to understand the mechanism of their effects.

### 5.2 *Deqi* and manipulations of acupuncture

*Deqi* is the key to the effectiveness of acupuncture (26), but *deqi* is a subjective feeling, difficult to quantify and evaluate, and currently lacks objective representation. The main purpose of fNIRS based research on *deqi* is to explore the brain response after acupuncture *deqi*, in order to provide objectified basis for acupuncture treatment. Simin Song discussed the effect of acupuncture *deqi* on the cerebral cortex of healthy people at *Shousanli* (LI10) acupoint, the result showed that *deqi* significantly activated the insular part of bilateral frontal lobes, DLPFC, the triangular part of the inferior frontal gyrus on the left side and the right FP region, thereby enhancing the brain FC between the ipsilateral parietal and temporal lobes, as well as between the left temporal lobe and the right parietal lobe (27). There was a correlation between *deqi* sensation and brain function response. Graph-theory analysis revealed that acupuncture at the *Shousanli* (LI10) acupoint with *deqi* activated the bilateral frontal lobes positively and enhanced FC among different brain regions, indicating that *deqi* at *Shousanli* (LI10) acupoint activated brain areas related to mental, thinking and cognitive functions. Another study explored the functional changes of the cerebral cortex after acupuncture *deqi* at *Hegu* (L14) acupoint (28). Researchers found that bilateral PFC and motor cortex (MC) were significantly inhibited during acupuncture manipulation. However, network connections with bilateral PFC as nodes showed a significant increase in FC during acupuncture manipulation. Meanwhile, *deqi* improved the network's efficiency and decreased shortest path length. Previous studies have shown that the MC had a role in pain control (29), and this study showed that acupuncture significantly improved the PFC-Motor connection, so acupuncture *deqi* could produce analgesic effects by increasing the FC of bilateral PFC.

Fernandez Rojas et al. (30) discovered that when performing needle insertion (NI) and needle removal (NR) at the *Hegu* (L14) acupoint, the hemodynamic activity of SI significantly increased. However, during rotation period, the hemodynamic activity decreased, which was believed to be possibly related to the subjects' familiarity with the sensation of manipulation and the analgesic effect exerted by the rotation operation of *Hegu* (L14) acupoint. In the experimental study, the hemodynamic responses after acupuncture manipulation showed strong activation and unique cortical networks in each stimulation, and the statistical results demonstrated significant differences.

The above research indicates that performing the acupuncture *deqi* operations at different acupoints triggered different cerebral cortex responses. The acupuncture *deqi* manipulations at the *Hegu* (L14) acupoint led to inhibition of hemodynamic in the frontal lobe cortex and MC of the brain, which might be related to the analgesic effect of acupuncture. Previous studies also supported that the acupuncture *deqi* at the *Hegu* (L14) acupoint had an anesthetic and pain-relieving effect (31).

### 5.3 Hyperscanning

Hyperscanning, as a new paradigm, has achieved the transformation from studying one person to studying “two or more people,” greatly strengthening the research on interpersonal brain mechanisms (32). The application of fNIRS hyperscanning in the study of interpersonal neural synchronization has been carried out in intimate relationships such as couples and parent-child relationships. These studies have found the phenomenon of robust and consistent interpersonal neural synchronization (INS) in the frontal, temporal and parietal regions of the brain between couples and lovers (33). In acupuncture research, hyperscanning studies could also be used to reveal interbrain neural synchronization in doctor-patient interaction, enhancing people's understanding of interpersonal neural synchronization.

One study took healthy people and acupuncturists as the research subjects. Among them, healthy people simulated patients and were randomly assigned to the verum acupuncture (VA) and sham acupuncture (SA) groups (34). Functional near-infrared spectroscopy hyperscanning was used to simultaneously record the neural responses of the “patient”- acupuncturist duo during the acupuncture stimulation in each group. The study found that in the VA group, The INS in the PFC of both the “patient” and the acupuncturist increased significantly during acupuncture, and was positively correlated with the acupuncture sensation of the patients. However, no significant neural synchronization phenomenon occurred in the SA group. Both the “patient” and the acupuncturist felt the difference in the strength of the acupuncture sensation between VA and SA during the acupuncture process. It was indicated that the PFC was very important for the interaction of the “patient”- acupuncturist binary group. The sensation of acupuncture might lead to an increase in INS between the “patient” and the acupuncturist. A previous fMRI study had demonstrated that during acupuncture treatment, there was an increase in INS between acupuncturists and patients, which correlates with enhanced therapeutic outcomes (35).

Traditional Chinese medicine holds that the interaction between doctors and patients is one of the important factors affecting the effect of acupuncture, which falls within the category of “treating the spirit” in traditional Chinese medicine. The above-mentioned hyperscanning study provided certain evidence from the perspective of imaging.

## 6 Acupuncture research of fNIRS under pathological condition

Acupuncture research under pathological conditions refers to exploring the impact of acupuncture intervention on the hemodynamics of patients' brains based on fNIRS. Through literature summary and analysis, it was found that its content mainly includes two dimensions: the immediate effect of acupuncture and the non-immediate effect of acupuncture. The types of diseases include insomnia, Mental disorders, Sequelae of stroke, disturbance of consciousness, Cognitive impairment and consciousness disorder, Tinnitus, Amblyopia, Parkinson's disease (PD).

### 6.1 Insomnia

Insomnia is also one of the common diseases in contemporary society. Acupuncture treatment has been proven to be an effective method for improving insomnia. fNIRS can reveal the therapeutic mechanism of acupuncture in alleviating insomnia by detecting the concentration of oxygenated hemoglobin in the cerebral cortex of patients with insomnia. Two fNIRS studies were conducted among patients with insomnia. One study explored the immediate effect of acupuncture on insomnia (36), while the other studied the cumulative effect of acupuncture on insomnia (37). Xu (36) discovered that acupuncture produced an immediate central effect on primary insomnia, specifically manifested as a decrease in the concentration of Oxy-Hb in brain regions such as the prefrontal motor cortex and the somatosensory synesthesia cortex of the parietal lobe, which inhibited excessive excitement in brain regions related to emotion and cognition, thereby controlling excessive arousal in insomniacs to improve insomnia. A longitudinal study investigated the changes in brain function of insomnia patients of different ages after acupuncture intervention (37). They used a facial emotion recognition task. They found that after acupuncture treatment, there were differences in the concentration of Oxy-Hb the frontal lobe among insomnia patients of different age groups under the stimulation of different attribute emotional faces. This indicated that the waxing and waning of “Yin” and “Yang” might be the reason for the differences in the therapeutic effects of acupuncture intervention in insomnia patients of different age groups. The above two studies indicate that acupuncture can regulate the oxygenated hemoglobin concentration in the frontal lobe and alleviate excessive arousal in patients with insomnia. Due to the differences in the research designs of the two studies and the varying ages of the subjects, there may be differences in the oxygenated hemoglobin concentration in the brain. The brain function mechanism by which acupuncture improves insomnia still requires further investigation.

### 6.2 Mental disorders

At present, acupuncture is regarded as an effective treatment for depression (38), anxiety disorders (39) and acute stress disorders (40), but there is no unified understanding of its therapeutic mechanism. Six studies explored the mechanism of acupuncture intervention in mental disorders. Four of them were longitudinal studies on the effect of acupuncture on depression (41–44), one was an immediate effect study on the intervention of acupuncture on anxiety disorders (45), and one was a longitudinal study on the intervention of acupuncture on acute stress disorders (46). The frontal cortex, particularly the DLPFC, is a crucial brain region for the generation and control of emotions (47, 48). The relevant research designated the prefrontal cortex as the target brain region. One study collected brain function data after acupuncture and western medicine intervention using an emotional picture task. The study found that after acupuncture intervention, the oxygenated hemoglobin concentration in the DLPFC of patients with depression increased (41). Another resting-state research found that acupuncture could increase the oxygenated hemoglobin level in the left DLPFC of patients with depression, and reduce the deoxygenated hemoglobin level (43). However, it had no significant effect on the left DLPFC of healthy subjects. This suggests that whether the brain is damaged or not is an important factor determining whether the relevant brain regions respond to acupuncture stimulation. The improvement of depressive symptoms by acupuncture may be achieved by activating the DLPFC on the left side, thereby enhancing the regulatory ability of the left dorsolateral prefrontal emotional center. In the study involving subjects with severe depression, the same emotional image task was used. The results showed that the severity of depression was negatively correlated with the blood oxygen saturation level in the prefrontal lobe (42). In another resting-state study, compared with the treatment with Western medicine alone, the combination of acupuncture and Western medicine was superior to the Western medicine treatment alone (44). The Resting-state functional connectivity (rsFC) of the DLPFC in the combined group was stronger, but no significant correlation was found between the changes in rsFC and the severity of depression.

Xianyin Xiang recruited patients with anxiety disorders to study the response of the cerebral cortex to acupuncture. Research (45) found that after a single acupuncture intervention, the hemoglobin concentration in the left DLPFC of patients with anxiety increased and the deoxygenated hemoglobin concentration decreased. However, healthy subjects did not show such reactions. This indicates that acupuncture intervention can immediately activate the left DLPFC of patients with anxiety disorders. The improvement of the mood of patients with anxiety disorders by acupuncture may be related to the activation of the left DLPFC.

In a randomized controlled crossover trial, the subjects were patients with acute disorders (46). To investigate the effects of true acupuncture and sham acupuncture combined with *Baihui* acupoint (GV20) on stress, the results showed that compared to sham acupuncture, true acupuncture combined with *Baihui* acupoint (GV20) not only could reduce the subjective stress level, but also could effectively promote the recovery of cerebral blood flow in the frontal lobe cortex.

The above research indicates that there are certain similarities in the changes of brain function in the DLPFC of the subjects after acupuncture intervention and multiple acupuncture interventions. Acupuncture may improve depression, anxiety disorders and acute stress disorders by activating the DLPFC. Moreover, fNIRS has certain potential in evaluating the severity of depression and the improvement of symptoms.

### 6.3 Sequelae of stroke

Stroke is a serious neurological disorder with high mortality and disability rates (49). In addition, there are also post-conditions such as movement disorders, swallowing difficulties and speech disorders. Acupuncture, including ear acupuncture and scalp acupuncture, is a common treatment method for stroke and its sequelae. At present, 8 fNIRS studies on acupuncture intervention for post-stroke sequelae have been carried out. Two of them are related to dysphagia (50, 51), five to movement disorders (52–56), and one to aphasia (57). Two studies examined the effect of acupuncture on brain function in patients with post-stroke dysphagia. Both trials involved conducting swallowing tests.

A study conducted by Ma (50) showed that patients with dysphagia have impaired activation of the swallowing cortex on the affected side, resulting in compensatory activity patterns on the healthy side. In the combined treatment group, after treatment, the FC between the middle prefrontal- left temporal-parietal complex, left prefrontal-right temporal-parietal complex, and left prefrontal-left temporal-parietal complex were significantly enhanced compared to the traditional rehabilitation group. This indicates that acupuncture treatment for dysphagia after stroke may achieve this through activating the swallowing cortex and reconstructing the swallowing brain network. Another experiment also found that the combined treatment group had better therapeutic effects than the traditional treatment group (51). The FC strength in the S1, M1, premotor cortex (PMC) and supplementary motor cortex (SMC) brain regions of the combined treatment group was higher. Based on the results of these two studies, it indicates that acupuncture may exert its effect by optimizing the brain network connections and promoting the remodeling of the cerebral cortex. Due to the fact that the affected brain regions by stroke are numerous, the healthy brain regions will compensate, so no definite conclusion can be drawn. The central mechanism of acupuncture intervention in post-stroke dysphagia still needs further exploration.

A randomized controlled study on aphasia after stroke used a resting-state setting (57). The results showed that the concentration of oxygenated hemoglobin in the left FP area, left fusiform gyrus, and left occipital part of the brain was increased in the acupuncture combined group. The concentration of oxygenated hemoglobin in the left FP area and the right superior temporal gyrus was significantly higher than that in the conventional treatment group. The changes in hemodynamic of the frontal-temporal brain regions might be the mechanism by which acupuncture takes effect.

Four randomized controlled trials investigated the effect of acupuncture on post-stroke motor disorders, including upper limb motor disorders, lower limb motor disorders, and hemiplegia. Zhang (53) used fNIRS to observe the effect of auricular acupuncture on the brain function of patients with upper limb motor disorders. In the study, the subjects were randomly divided into the real auricular acupuncture group and the sham auricular acupuncture group. After the experiment, it was found that compared with the sham auricular acupuncture group, the peak value of the motor evoked potential (MEP) in the M1 of the brain in the real auricular acupuncture group significantly increased, and the oxygenated hemoglobin content also significantly increased, showing a significant activation response. The upper limb motor function deficits of the patients in the real auricular acupuncture group were significantly improved, suggesting that the activation of the brain's M1 area might be the mechanism by which auricular acupuncture improves upper limb motor dysfunction after stroke. Another study recruited stroke patients with lower limb motor disorders and used acupuncture combined with rehabilitation therapy as the intervention method (52). By comparing the fNIRS detection data before and after the intervention, it was found that the FC of the PMC in the damaged hemisphere of the subjects increased, and the asymmetric activation state of the sensory-motor cortex was also improved. The correlation analysis indicated that the changes in lower limb motor function scores are positively correlated with the changes in the FC strength of the PMC. Their research results indicated that acupuncture may improve lower limb motor disorders by enhancing the activation function of the contralateral PMC. In order to explore the efficacy and mechanism of acupuncture combined with hydrotherapy in improving the condition of cerebral infarction, a resting-state research trial (54) revealed that acupuncture combined with hydrotherapy could significantly enhance the activation of the M1 area on the affected side of the patients, activate the brain nerve functions, and improve the motor functions. Based on the patient's muscle tone and the onset time, the rehabilitation treatment after stroke can be divided into three stages: the flaccid stage, the spastic stage, and the recovery stage. The body functions of patients in these three stages are in different states, and the acupuncture regimens are not completely the same. A study was conducted to observe the differences in the therapeutic effects of different acupuncture regimens at different stages of stroke and the same acupuncture regimens (55). They used fNIRS to detect the differences in brain function between the two groups of subjects before and after the intervention. The study found that the activation degree of the M1 brain region in the staged acupuncture group was significantly higher than that in the unified acupuncture group, and the clinical efficacy was also better than that of the unified acupuncture group. Their results indicate that the staged acupuncture therapy is more conducive to the recovery of hemiplegic patients and also increases the activation intensity of the M1.

A study observed the changes in the cerebral cortex of hemiplegic patients with immediate acupuncture after stroke (56). Based on the severity of the motor dysfunction of the subjects, the researchers divided the patients into a mild injury group and a severe injury group. The research results confirmed that acupuncture therapy could induce bilateral brain activation responses in patients with hemiplegic stroke. Under the effect of acupuncture, the activation responses of the cerebral cortex in the severe injury group were more significant than those in the mild injury group, and were particularly evident in the damaged hemisphere area.

Based on the above research results, it can be concluded that acupuncture (scalp acupuncture, auricular acupuncture) can improve motor dysfunction after stroke, enhance the activation of the MC and FC. The trial protocols and the affected limb sites of the subjects in the five studies were different, and there were also differences in the types of acupuncture. Therefore, no definite conclusion could be drawn. The mechanism of acupuncture in treating motor disorders after stroke still needs further exploration.

### 6.4 Disorders of consciousness

Two fNIRS studies on consciousness disorders both examined the effect of acupuncture on the functional activity of the PFC. However, the design of the two studies was different. One randomized controlled trial compared a single verum acupuncture and a SA session (58). The results showed that the activation level of bilateral DLPFC in the acupuncture group was significantly higher than that in the SA group. The resting-state FC between DLPFC-M1, DLPFC-M1, and S1-S1 brain regions was enhanced, but no significant correlation was found between the level of consciousness and the activation response/function connection. Another study observed the impact of acupuncture on patients with different degrees of consciousness disorders. Their research found that the PFC was activated during acupuncture manipulation, and FC was enhanced. However, the connection strength of the left cerebral cortex was generally higher than that of the right cerebral cortex. There was no significant difference in hemodynamics between the vegetative state (VS) and the minimally conscious state (MCS) groups (59). The above research results suggest that the activation of prefrontal lobe brain function and the enhancement of FC may indicate the central mechanism of acupuncture in treating disorders of consciousness.

### 6.5 Mild cognitive impairment (MCI)

Cognition refers to the process by which people acquire or apply knowledge, or the process of information processing. It encompasses sensation, perception, memory, thinking, imagination and language. Normal cognitive function depends on the normal operation of neural function networks in different brain regions. Two studies attempted to explore the brain functional mechanism of acupuncture intervention for MCI through fNIRS (60, 61). Both studies employed a working memory task and focused on the prefrontal lobe as the interest brain region. One study found that after 12 weeks of acupuncture intervention, the cognitive function of MCI patients improved, and the prefrontal cortex was also significantly activated, and the brain functional connections were also enhanced (60). The other study collected fNIRS data from MCI and healthy subjects before acupuncture intervention, conducted machine learning, and established a classification model (61). After acupuncture intervention, discriminant analysis was performed on the fNIRS data collected from MCI patients at different treatment stages. It was found that as the cycle lengthened, the accuracy of the classification model continuously decreased, indicating that the brain responses of the patients improved. The above two studies collectively indicate that the central mechanism by which acupuncture improves MCI may be related to the activation of the PFC brain region and the enhancement of FC. The use of fNIRS combined with machine learning has certain potential in the application of MCI diagnosis.

### 6.6 Tinnitus

Although human research on the neural mechanism of tinnitus has been carried out for several decades, it is still unclear at present. In a study exploring the mechanism of acupuncture intervention in tinnitus, they recruited 18 patients with subjective tinnitus who met the criteria and gave them 10 sessions of acupuncture treatment (62). fNIRS detection was performed during sound-induced activities before and after the treatment. The results showed that after acupuncture intervention, the concentration of Oxy-Hb in the temporal lobe of tinnitus patients increased, the temporal auditory cortex was significantly activated, and the scores of related hearing impairment scales decreased significantly. Another fNIRS study also found on the hemodynamic changes of the temporal lobe in healthy subjects and tinnitus patients under the sound stimulation task that acupuncture could significantly increase the blood oxygen concentration in the temporal lobe of tinnitus subjects, and the blood oxygen concentration level was in a favorable relationship with the hearing impairment scale (63). The above research results indicate that acupuncture intervention for tinnitus exerts therapeutic effects by increasing the blood oxygen level in the temporal lobe and activating the temporal auditory cortex.

### 6.7 Amblyopia

Amblyopia is a common and frequently-occurring disease among teenagers, which has a huge impact on the daily life of patients (64). Existing studies have proved that acupuncture may exert its effect by regulating the function of the anterior cerebellar brain region (65). Through the search, a total of two studies on fNIRS-based acupuncture intervention for amblyopia were identified. Both studies employed visual tasks to assess the activation levels of brain regions.

A study on amblyopia found that the activation range of the visual cortex in patients with amblyopia was smaller than that in healthy subjects, indicating that there might be dysfunction in the visual cortex function of children with amblyopia (66). Acupuncture combined with Western medicine treatment could improve the vision of patients with amblyopia more effectively than Western medicine treatment alone. The mechanism was related to the fact that acupuncture increases the blood flow in the occipital region of patients and improved the hemodynamic response of the visual cortex. Another study randomly divided the amblyopia subjects into the amblyopia group, the traditional acupuncture group, and the percutaneous electrical acupoint stimulation group (67). fNIRS detection was conducted before and after the intervention, respectively. The study found that when one eye received visual stimulation, both the primary visual cortex and the secondary visual cortex of children with anisometropic amblyopia had blood oxygen dysfunction, and there was asymmetric involvement between the bilateral visual cortices. Both traditional acupuncture and percutaneous electrical acupoint stimulation intervention could increase the Oxy-Hb content in the visual cortex of children with anisometropic amblyopia when they receive visual stimulation in one eye, and at the same time improved the asymmetric involvement between the ipsilateral and contralateral visual cortices. However, traditional acupuncture had more advantages in the intervention of anisometropic amblyopia.

### 6.8 PD

As a neurological disease with pathological tremor as the main symptom, the occurrence of tremor in PD is closely related to the MC (68). The movement disorder caused by tremor seriously reduces the quality of life of PD, while acupuncture has been proven to improve the motor function of PD (69), but the mechanism is still unclear. A randomized controlled trial of fNIRS for acupuncture intervention in PD proved that acupuncture could improve gait disorders in PD patients by increasing their stride length, single support times (70). fNIRS detection proved that it exerted its effect by increasing the concentration of Oxy-Hb in the PFC and auxiliary motor area. This provided new ideas for the mechanism of acupuncture in the treatment of PD.

## 7 Application of fNIRS in animal acupuncture research

At present, there were relatively few animal experimental literatures on the application of fNIRS in acupuncture. The author has searched out a total of 4. A study utilized fNIRS to investigate the brain functional mechanism of acupuncture intervention in post-traumatic stress disorder (PTSD) (71). This experiment took PTSD model rats as the experimental subjects, and used acupoints such as *Baihui* (GV20), *Shenmen* (PC6), *Neiguan* (HT7), and *Taizhong* (LR3) as the stimulation points to detect the changes in blood oxygen concentration in the cerebral cortex of the rats before and after the intervention. fNIRS data showed that compared with the blank control group, before acupuncture intervention, the concentration of Oxy-Hb and Total-Hb in brain regions such as the frontal lobe, hippocampus, and amygdala of rats increased, while the concentration of Deoxy-Hb decreased. After the intervention, in the acupuncture group, the concentration of Oxy-Hb and Total-Hb in brain regions such as the frontal lobe, hippocampus and amygdala were significantly decreased, Deoxy-Hb was significantly increased. The research results indicated that acupuncture can alleviate the anxiety and depression symptoms in rats. The mechanism may be achieved by inhibiting abnormal cortical activities in brain regions such as the prefrontal lobe, hippocampus, and amygdala, stabilizing the energy metabolism requirements of cells, and restoring the normal oxygen consumption in related brain regions.

To explore the brain functional mechanism of acupuncture in the treatment of amblyopia, a study established a rat model of monocular amblyopia using the right single eyelid suture method, compared the efficacy of acupuncture and levodopmethyl in the treatment of amblyopia, and used fNIRS to detect the changes in cerebral blood flow in the visual cortex of rats (72). It was found that acupuncture improved the phenomena of prolonged latency and reduced amplitude of visual evoked potential in amblyopic rats. At the same time, it was found that the content of Oxy-Hb in the ocular dominant column area of the visual cortex of amblyopic eyes significantly decreased after modeling. Both acupuncture and drug treatment increased the content of Oxy-Hb in this area. It was indicated that both acupuncture and drugs can regulate the abnormal functions of the key areas of the visual cortex in amblyopia and adjust the changes of visual electrophysiological plasticity to improve amblyopia.

fNIRS has also been applied in the research of animal insomnia models with acupuncture intervention. An experimental study based on the “neurovascular coupling mechanism” proved that both acupuncture and estazolam could significantly improve the cognitive functions such as mood and learning in insomniacs, but the regulatory effect of acupuncture on anxiety and depression caused by insomnia was better than that of estazolam (73). fNIRS detection revealed that before treatment, the concentrations of Deoxy-Hb in the prefrontal lobe and occipital lobe were significantly decreased, while the concentrations of oxygenated hemoglobin and total hemoglobin were significantly increased. After treatment, the concentration of deoxyhemoglobin was significantly increased, while the concentrations of Oxy-Hb and Total-Hb were significantly decreased. The mechanism by which acupuncture improve insomnia may be related to the regulation of blood oxygen metabolism disorders in the prefrontal lobe and occipital lobe by acupuncture.

Another animal experiment randomly divided Alzheimer's disease (AD) model rats into the model group, the donepezil hydrochloride group and the acupuncture group (74). There were 10 in each group. After the treatment, the changes of Oxy-Hb, Deoxy-Hb and Total-Hb in the cerebral cortex of rats in each group were detected by fNIRs. The results showed that the escape latency was shortened in both the donepezil hydrochloride group and the acupuncture group, suggesting that acupuncture of the “Yizhi Tiaoshen” acupoint formula and oral administration of donepezil hydrochloride can improve the learning and memory function of AD rats. The fNIRS test results indicated that Oxy-Hb and Total-Hb increased in the prefrontal lobe and parietal lobe after the intervention, while the concentration of Deoxy-Hb decreased. It was indicated that acupuncture improve insomnia in AD rats by activating the prefrontal lobe and parietal lobe and increasing the blood oxygen metabolism level in the prefrontal lobe and parietal lobe regions.

In conclusion, whether it was PTSD, amblyopia, insomnia or AD, acupuncture can exert its effect by adjusting the blood oxygen metabolism in the corresponding brain regions and improving the abnormal functional activities of the related brain regions. Among them, the improvement of symptoms in rats with post-traumatic stress disorder was related to the inhibition of functional activities in brain regions such as the prefrontal lobe, hippocampus, and amygdala. The mechanism by which acupuncture improve amblyopia may be generated by increasing the concentration of oxygenated hemoglobin in the visual cortex to stimulate and regulate abnormal activities in key areas of the visual cortex. Acupuncture intervention for AD worked by improving blood oxygen metabolism in the prefrontal and parietal lobes and protecting neuronal function. Acupuncture improve insomnia by inhibiting abnormal functional activities in brain regions such as the prefrontal lobe and occipital lobe and reducing blood oxygen metabolism. These animal experiments provided new basis for the clinical practice and research of acupuncture.

## 8 Discussion

This study analyzed and summarized the specific applications of fNIRS in the field of acupuncture research in recent years. A total of 35 studies were retrieved, including four animal experiments and 31 human experiments, among which 10 were randomized controlled trials. Most of the research was on the mechanism of the therapeutic effect of acupuncture, while a small part was on the theoretical mechanisms of acupuncture such as reinforcing-reducing manipulation and deqi. The main types of diseases studied included depression, anxiety, insomnia, stroke, MCI, AD, consciousness disorders, traumatic stress disorder, PD, tinnitus, amblyopia, etc. Most of these diseases belonged to the category of brain function disorders.

The design paradigm of acupuncture research under physiological conditions was to needle specific acupoints. The changes in cerebral cortex hemodynamics before and after the intervention were detected by fNIRS. The research content was closely related to the basic theories of acupuncture, such as ARRM and acupuncture *deqi*. For acupuncture studies under pathological conditions, most of the research design paradigms took the hemodynamic data of the brain of healthy people or before acupuncture intervention as the baseline standard, stimulated the relevant acupoints, and then conducted fNIRS detection immediately after acupuncture intervention or after multiple acupuncture interventions. The purpose was to explore the immediate therapeutic effect and cumulative effect of acupuncture on related diseases. The animal experiment design model took disease-modeled rats as the experimental subjects, with Western medicine intervention as the control. fNIRS detection was conducted before and after the acupuncture intervention to explore the therapeutic mechanism of acupuncture. Because the rat brain is small in size, the detection depth of fNIRS was not limited to the cerebral cortex but also included deep brain regions. Therefore, its detection was more comprehensive and has certain advantages compared with human studies.

In the study of ARRM, through comparative analysis, we found that the PFC, M1, and S1 might be the key brain regions where ARRM take effect. Given the scarcity of research literature on ARRMs and the fact that the results of the two literatures included in this article were not all the same, it was still impossible to draw a definite conclusion. In the research related to the operation of acupuncture *deqi*, the most commonly used acupoint was *Hegu* acupoint. By analyzing these research results, we found that obtaining qi through acupuncture increased the FC between the frontal lobes. *Deqi* was correlated with brain functional responses. The changes in cerebral hemodynamics caused by acupuncture *deqi* may be the basis for the analgesic effect of acupuncture. The results of the hyperscan study between doctors and patients showed that the prefrontal lobe was the target brain region of INS for both doctors and patients, and this increase in INS was positively correlated with needle sensation.

In the fNIRS studies under pathological conditions, for diseases such as insomnia, anxiety, and stroke, the relevant brain regions of the cerebral cortex were activated immediately after acupuncture intervention. For instance, in two studies on consciousness disorders, the results both showed that a single acupuncture session immediately activated the DLPFC and M1 related to consciousness, and these brain regions were closely related to consciousness. This provided sufficient basis for the immediate mechanism of action of acupuncture. Acupuncture studies combining non-immediate effects have found that for the same disease, both immediate and non-immediate acupuncture could activate the functional activities of the same brain regions and increase the FC of related brain regions. For example, in insomnia, both immediate and non-immediate acupuncture could reduce the hemodynamics of the DLPFC related to controlling arousal. It indicated that whether it was the cumulative effect of multiple acupuncture or the immediate effect of single acupuncture intervention, the cerebral cortex response brain regions were the same, proving that the reduction of blood oxygen concentration in the prefrontal lobe may be one of the mechanisms by which acupuncture improved insomnia. For chronic diseases such as insomnia, stable and long-lasting clinical efficacy could only be achieved after multiple acupuncture stimulations. In limb movement disorders caused by stroke, we found that acupuncture activated brain regions such as PMC, M1, and SMC and strengthened the functional connections between brain regions, thereby exerting its effect. For PD, acupuncture could activate the PFC and the auxiliary motor area to exert therapeutic effects. For mental disorders such as anxiety and depression, acupuncture was effective in improving related symptoms by activating the PFC, especially the DLPFC. Patients with amblyopia have disorders in the blood oxygen concentration of the visual cortex. Acupuncture exerted its therapeutic effect by increasing the local blood oxygen concentration. The treatment of cognitive dysfunction by acupuncture worked by activating the blood oxygen concentration in the PFC and increasing the FC between brain regions. Tinnitus studies have confirmed that acupuncture worked by activating the temporal lobe.

Animal experimental studies have provided the possibility for some clinical studies that are impossible or difficult to carry out. The results have further verified the clinical research results. Among them, acupuncture intervention for insomnia was not only related to the PFC but also closely related to the occipital lobe, which was not entirely the same as the clinical research results. This suggests that in future studies, a combination of animal and human clinical research can be adopted. To provide more reliable research results.

## 9 Limitations and future research directions

Although fNIRS has made certain progress in acupuncture research, there are still some problems. Firstly, fNIRS detection lacks standardization. Currently, compared with fMRI, EEG and other techniques, fNIRS technology is still not mature enough. In different studies, the types of fNIRS instruments, the number of probes and their placement positions are all different. Even for the same disease, such as insomnia, the fNIRS task setting type, frequency, and covered brain regions are different, which may affect the accuracy of the test results. Secondly, the research direction is relatively limited. fNIRS mainly focuses on clinical trials of acupuncture, while there are relatively few studies related to animal experiments of acupuncture and the mechanism of acupuncture. And most of the diseases studied are brain dysfunction diseases. Finally, the quality of the research remains uneven. Currently, the overall number of acupuncture fNIRS studies is still relatively small, and RCT trials are even fewer. And most of the fNIRS studies involving acupuncture have been conducted in Asian countries such as China, Japan and South Korea, while fewer such studies have been carried out in other countries. Moreover, the sample size in various studies is mostly 10 to 30 cases, which is relatively small. Even for the same disease in different studies, the age and gender of the included subjects vary greatly, which will cause significant differences among the research results. This leads to the difficulty in widely promoting the research results.

In the future, improvements should be made in the following directions: (1) standardize the fNIRS detection technology and plan. When conducting research and design, factors such as the model and parameters of the fNIRS machine, the number of probes, and the placement of brain regions should be taken into account. (2) Expand research types, such as conducting more research on acupuncture theories (such as acupuncture analgesia, acupuncture anesthesia, acupuncture qi acquisition and acupuncture techniques, etc.), animal experiments and studies on diseases other than brain diseases. (3) Optimize the research design to improve the research quality, such as conducting more RCT trials, expanding the sample size in the trials, and strictly adhering to the inclusion criteria for subjects, etc. (4) We look forward to more non-Asian countries conducting more fNIRS studies on acupuncture.

## 10 Conclusion

As an emerging imaging technique, fNIRS has a high spatiotemporal resolution and has opened up a new visualization approach for the study of acupuncture theory and therapeutic mechanisms. Although certain research achievements have been made in acupuncture manipulation, acupuncture *deqi* and acupuncture intervention for brain dysfunction diseases at present, in the future, it is still necessary to take advantage of the fNIRS technology to carry out high-quality research to reveal the scientific principles of acupuncture.
