# Supplementary material for: Analysis of the application of functional near-infrared spectroscopy in acupuncture research: a review
Source: Front Neurol. 2025 Sep 19;16:1644010. doi: 10.3389/fneur.2025.1644010 (PMC12491014; doi:10.3389/fneur.2025.1644010)
Supplement: Supplementary file 1 [file Data_Sheet_1.doc]

**Supplementary Table1. Search strategy for each database**

| **Databases** | **Search strategy** |
| --- | --- |
| PubMed | ("Functional Near-Infrared Spectroscopy"[Title/Abstract] OR fNIRS[Title/Abstract]) AND (Acupuncture[Title/Abstract] ) |
| Web of Science | TS=("Functional Near-Infrared Spectroscopy" OR fNIRS ) AND TS=(Acupuncture) |
| China National Knowledge Infrastructure (CNKI) | ("Functional Near-Infrared Spectroscopy"[Title/Abstract] OR fNIRS[Title/Abstract]) AND (Acupuncture[Title/Abstract] ) |
| Wanfang Database | ("Functional Near-Infrared Spectroscopy"[Title/Abstract] OR fNIRS[Title/Abstract]) AND (Acupuncture[Title/Abstract] ) |
| China Science and Technology Journal Database(VIP) | ("Functional Near-Infrared Spectroscopy"[Title/Abstract] OR fNIRS[Title/Abstract]) AND (Acupuncture[Title/Abstract] ) |

**1.1.** Search strategy in PubMed databas

| No. Search items |
| --- |
| #1 Acupuncture. Mesh.  #2 Acupuncture therapy. ti. ab.  #3 Acupuncture points. ti. ab.  #4 Manual acupuncture. ti. ab.  #5 Auricular acupuncture. ti. ab.  #6 Scalp acupuncture. ti. ab.  #7 Body acupuncture. ti. ab.  #8 1 or 2-7  #9 Functional Near-Infrared Spectroscopy. Mesh.  #10 fNIRS. ti. ab.  #11 9 or 10  #12 #8 and #11 |

**1.2.** Search strategy in Web of Science

| No. Search items |
| --- |
| #1 Topic:(“Acupuncture” OR “Acupuncture therapy” OR “Acupuncturepoints” OR “Manual acupuncture” OR “Auricular acupuncture” OR “Auricular acupressure” OR “Scalp acupuncture” OR “Body acupuncture”  #2 “Functional Near-Infrared Spectroscopy” OR “fNIRS”  #3 #1 AND #2 |

**1.3.** Search strategy in China National Knowledge Infrastructure Database

| No. Search items |
| --- |
| #1 SU=(针刺'+'耳针'+'头针'+'手针'+'体针')  #2 SU='功能性近红外光谱技术'  #3 #1 AND # 2 |

**1.4.** Search strategy in Wan Fang Data

| No. Search items |
| --- |
| #1 主题=(针刺or耳针or头针or手针or 体针)  #2 主题=(功能性近红外光谱技术)  #3 #1 AND # 2 |

**1.5.** Search strategy in Chinese Science and Technology Periodical Database(VIP)

| No. Search items |
| --- |
| #1 M=(针刺or耳针or头针or手针or 体针)  #2 M=(功能性近红外光谱技术)  #3 #1 AND # 2 |

**Supplementary Table 2. Inclusion and Exclusion Criteria for Literature**

| **Inclusion Criteria** | **Exclusion Criteria** |
| --- | --- |
| 1. Primary research articles, original studies, using near-infrared spectroscopy to investigate any aspect of the effect or mechanism of acupuncture.. 2. Studies conducted in humans or rats 3. Publications published in English or Chinese | 1. References such as case reports, reviews and trial protocols 2. The intervention method is not acupuncture or acupuncture combined with others. 3. Publish in non-English or Chinese language |
